# The relationship between comorbidity medication adherence and health related quality of life among patients with cancer

**DOI:** 10.1186/s41687-018-0057-2

**Published:** 2018-07-04

**Authors:** Dana Drzayich Antol, Adrianne Waldman Casebeer, Raya Khoury, Todd Michael, Andrew Renda, Sari Hopson, Aparna Parikh, Alisha Stein, Mary Costantino, Stephen Stemkowski, Mikele Bunce

**Affiliations:** 1 Louisville, Kentucky USA; 2 South San Francisco, California USA; 3 Humana Inc, Louisville, Kentucky USA

**Keywords:** Health-related quality of life, Healthy days, Cancer, Comorbidities, Medication adherence

## Abstract

**Background:**

Studies have demonstrated that comorbidities compound the adverse influence of cancer on health-related quality of life (HRQoL). Comorbidities adversely impact adherence to cancer treatment. Additionally, adherence to medications for comorbidities is positively associated with HRQoL for various diseases. This study used the Center for Disease Control and Prevention’s Healthy Days measure of HRQoL to explore the association between HRQoL and adherence to comorbidity medication for elderly patients with cancer and at least one comorbid condition.

**Methods:**

We conducted a cross-sectional survey combined with retrospective claims data. Patients with metastatic breast, lung or colorectal cancer were surveyed regarding their HRQoL, comorbidity medication adherence and cancer-related symptoms. Patients reported the number of physical, mental and total unhealthy days in the prior month. The Morisky Medication Adherence 8-point scale was differentiated into moderate/high (> 6) and low (≤ 6) comorbidity medication adherence.

**Results:**

Of the 1847 respondents, the mean age was 69.2 years, most were female (66.8%) and the majority of the sample had Medicare coverage (88.2%). Low comorbidity medication adherence was associated with significantly more total, mental and physical unhealthy days. Low comorbidity medication adherence was associated with the presence of patient-reported cancer-related symptoms. Patients reporting low, as compared to moderate/high, comorbidity medication adherence had 23.4% more unhealthy days in adjusted analysis, *P* = 0.007.

**Conclusion:**

The positive association between low comorbidity medication adherence and the number of unhealthy days suggests that addressing barriers to comorbidity medication adherence during cancer treatment may be an avenue for improving or maintaining HRQoL for older patients with cancer and comorbid conditions.

## Background

Patients with cancer have high rates of comorbid conditions, some of which may be age related [1, 2]. Nearly 70% of patients with cancer have one comorbid condition, and more than 30% of patients with cancer had two or more comorbidities [2]. Studies have also demonstrated that patients with cancer and comorbidities have lower survival rates than patients with no other comorbidities [3–8]. Specifically, Jørgensen et al. (2012) [5] found higher overall mortality rates in elderly patients with lung, colorectal, and prostate cancer and higher comorbidity levels. The additional disease burden for patients with both cancer and other comorbidities is compounded by the challenges of managing maintenance pharmacologic treatment for these comorbidities while simultaneously undergoing cancer treatment.

Higher levels of adherence to medications addressing comorbidities have been found to be predictive of better health related quality of life (HRQoL) in other disease states. For example, adherence to comorbidity medications has been found to be predictive of improved medical outcomes for patients with diabetes [9]. Poor adherence to medication has been identified as the key contributing factor to failed hypertension control [10]. For example, Bansilal et al. (2016) [11] evaluated the association between either partial medication adherence or nonadherence compared to full adherence among patients hospitalized for a myocardial infarction or atherosclerotic disease. Compared to patients adherent to their medication, patients partially adherent had a 19% increased risk of a major adverse cardiovascular event and patients who were nonadherent had a 27% increased risk of mortality. For patients with cancer, studies of medication adherence have generally focused on compliance to treatment, and have shown that comorbid conditions reduce follow through with cancer treatment [12]. Studies of the influence of adherence to comorbidity medications and HRQoL for patients with cancer have been lacking, and may provide an opportunity for improving patients’ HRQoL.

The Centers for Disease Control and Preventions (CDC) Healthy Days measure is a tool for measuring HRQoL. The core module of this simple yet comprehensive tool, which has been included in the Behavioral Risk Factor Surveillance System since 1993, includes four questions on general health, physical unhealthy days, mental unhealthy days and activity limitations [13]. Of particular interest and relevance, the tool gives equal weight to physical and mental health when assessing HRQoL. The Healthy Days measure has been used in clinical, public health and research settings, and has been correlated with disease progression and health outcomes [14–18]. The Healthy Days measure has been compared to other commonly used tools to assess HRQoL, including the SF-36 [19] and the PROMIS Global Health Scale [20], and has been shown to compare favorably to these tools, while using fewer questions.

Given the positive relationship between adherence to medications and HRQoL and the high prevalence of comorbidities among patients with cancer, the purpose of this study was to investigate the influence of adherence to medications for comorbidities on HRQoL for patients with cancer. Additionally, this study sought to utilize a validated and parsimonious measure of HRQoL in a cohort of patients with cancer. Specifically, this study evaluated the hypothesis that the number of unhealthy days a patient with metastatic cancer experiences would be adversely affected by low adherence to comorbidity medication. We further hypothesized that nonadherence to comorbidity medication would be associated with an increase in cancer-related symptoms. The overarching goal is to identify actionable opportunities to assess and improve HRQoL for older patients with cancer ad comorbid conditions.

## Methods

### Data source

The data for this study were obtained from a cross-sectional survey of patients with metastatic cancer merged with administrative claims data from Humana Inc., a health and wellness company. The mailed survey included validated patient-reported measures of HRQoL, as well as other patient reported outcomes. The administrative claims data included enrollment, medical, and pharmacy data. The research protocol was approved by an independent institutional review board.

### Patients

While a majority of patients enrolled in a Medicare Advantage plan, patients dually enrolled in Medicare and Medicaid or enrolled in a commercial health plan were considered for this study. Humana administers government funded health insurance for the elderly (age 65 and older) and patients with select disabilities through the Medicare program and health insurance for the impoverished through the Medicaid program. Humana commercial health plans are largely employer-sponsored benefits. Adults (19–89 years) with metastatic breast, lung, or colorectal cancer who received radiotherapy, chemotherapy, or surgical treatment for their cancer between January 1, 2014 and March 31, 2015 (identification period) were considered for inclusion in this study. Metastatic status was defined as having two or more International Classification of Disease, Ninth Revision, Clinical Modification (ICD-9-CM) diagnostic codes for metastatic disease (196.0×-196.1×, 196.3×-196.5×, 196.8×, 197.0×-197.3×, 197.xx, 198.xx) occurring on separate days, between 180 days prior to and up to 120 days after the first treatment date. Primary cancers associated with the metastatic codes were defined as two or more primary cancer ICD-9-CM codes [breast (174.xx), colorectal (153.xx, 154.xx), lung (162.2× – 162.9×, 163.xx)] within a 2 year period, at least 30 days apart or 90 days after the first metastatic code. The index date was defined as the earlier of two independent ICD-9-CM codes for chemotherapy or two independent codes for radiotherapy, or one code for cancer-specific surgery accompanied by at least one code for radiotherapy or chemotherapy within the identification period. If additional primary cancers (defined as two separate diagnoses of a cancer other than the cancer types of interest) were found in the pre-index period, the patient was excluded from the study.

The pre-index period was defined as 2 years prior to the date of the metastatic disease diagnosis. The index date was based on two qualifying claims for chemotherapy, two qualifying claims for radiotherapy, or one code for cancer-specific surgery accompanied by one claim for radiotherapy or chemotherapy. Qualifying chemotherapy claims were based on National Comprehensive Cancer Network guideline recommended oncology medications from either the medical or pharmacy claims for the specific cancer type [19, 21, 22]. Treatment had to have occurred within 90 days of survey administration to ascertain if the patient was currently undergoing treatment at the time the survey was completed.

Patients were required to be enrolled in a Humana health plan at the time the survey was administered and for 2 years preceding the survey. Indication of one or more pre-index comorbidities, for which maintenance medications were likely to have been prescribed, was also required for inclusion in the study. The qualifying comorbidities were identified in administrative claims data by ICD-9-CM codes, and included, for example, cerebrovascular disease, diabetes mellitus and depression. The full list of qualifying comorbidities and their respective ICD-9-CM codes can be found in Appendix 1.

The mailed survey was administered to 7432 eligible patients with a completed survey returned by 32% (*N* = 2389) of recipients. Post-hoc exclusions were applied for patients who indicated on the survey that they had not received cancer treatment within the prior 3 months, were not enrolled in a Humana health plan at the time of survey completion or who were deceased as of the date of survey administration, since the survey may have been completed by a household member or caregiver. The final study cohort was comprised of 524 patients with metastatic colorectal cancer, 623 with metastatic lung cancer, and 700 with metastatic breast cancer for a total of 1847 evaluable patients. The Healthy Days survey tool was complete for 93.4% (*n* = 1780) of the survey respondents and the comorbidity medication adherence scale for 93.1% (*n* = 1720).

### Measures

#### Patient-reported measures

##### Sociodemographic characteristics

Sociodemographic factors assessed for differences in comorbidity medication adherence included: marital status, and living status, which was classified as living alone versus living with others.

##### Healthy days

HRQoL was evaluated using the four-item Healthy Days Core Module (Appendix 2), developed by the Centers for Disease Control and Prevention. Patients were asked discrete questions to quantify the number of days in the last 30 days that either their physical or mental health was not good. Individually, these questions provided the number of physical and mental unhealthy days. The number of days is summed to a maximum of 30 to create a measure of the total number of unhealthy days a patient has experienced in the last month. To indicate those patients who had frequent health distress, the total number of unhealthy days was dichotomized with ≥14 total unhealthy days considered frequent unhealthy days. This same cut-off was applied to the number of physical and mental unhealthy days. Patients also rated their overall health as excellent, very good, good, fair or poor, and indicated the number of days in the last 30 days that their physical or mental health kept them from their usual activities.

##### Comorbidity medication adherence

The Morisky Medication Adherence Scale (MMAS, Appendix 3) was used to ascertain patient-reported adherence to comorbidity medications [23]. The eight-item MMAS is typically used to assess adherence to a specific medication and includes questions regarding the patient’s specific adherence behaviors and knowledge regarding their prescriptions. This measure was selected because of its ability to distinguish barriers to adherence as well as medication consumption as compared to a claims-based measure which can only assess medication fills. The MMAS is strongly associated with claims-based measures of medication adherence [24]. In this survey, patients were asked to recall adherence to medications related to the management of their comorbid conditions. A summary score was calculated for each patient based on the number of positive responses. The scores range from zero to eight with low comorbidity medication adherence defined as scores less than six and medium/high comorbidity medication adherence defined as scores greater than or equal to six [23].

##### Cancer-related symptoms

Recognizing that symptoms related to metastatic cancer and side-effects from cancer treatment may not be adequately identified in claims data, the survey captured patient-reported cancer-related symptoms and side-effects. This study considered cancer-related symptoms as either manifestations of cancer or cancer treatment collectively, acknowledging that the cause of the symptom(s) is not always discernable, particularly to the patient. Questions ascertaining cancer-related symptoms were modeled after questions on the Medical Health Outcomes Study (MHOS) [25] and captured the frequency of symptoms as never, rarely, sometimes, often or always. Cancer-related symptoms evaluated included pain, fatigue, nausea/vomiting, diarrhea and shortness of breath. For analysis, patient-reported symptoms rated as sometimes/often/always were considered present and those rated as never/rarely were considered absent.

#### Claims-based measures

##### Sociodemographic characteristics

Sociodemographic characteristics derived from claims data included: patient age, sex, race for patients with Medicare, and geographic region based on the patient’s state of residence on the index date. States were classified in regions based on census classifications. Additionally, Medicare versus commercial coverage, and dual eligibility (defined as Medicare members who are also eligible for Medicaid) were obtained from administrative claims enrollment data. Dual-eligibility for both the Medicaid and Medicare programs is suggestive of patient disability.

##### Comorbidities

The level of patient comorbidity was evaluated for its relationship with the degree of comorbidity medication adherence. This was measured using the Deyo-Charlson Comorbidity Index (DCCI), a measure that uses health insurance claims and 17 categories of comorbidity to calculate a score, ranging from zero to six, that reflects the probability of one-year mortality [26]. In this study, we considered comorbidity in the 6 months prior to the index date; the Klabunde modification of the DCCI was used since it includes comorbidities from both the hospital and physician office settings [27]. Based on the distribution of the data, comorbidity scores were dichotomized with low comorbidity level defined as a score less than seven and moderate/high comorbidity level defined as scores greater than or equal to seven. Individual comorbidities used to calculate the DCCI with prevalence of 10% or greater were reported separately.

### Statistical analysis

The central tendency of continuous variables was reported as means (standard deviation) for data approximating a normal distribution and as medians [interquartile range] for skewed data. Statistical significance was assessed for categorical variables using chi-square tests, and t-tests for continuous variables. The median number of unhealthy days was compared by level of comorbidity medication adherence using the Wilcoxon rank sum test.

A multivariable negative binomial regression model was used to establish the association between low comorbidity medication adherence and the number of unhealthy days. Other covariates included in the model were: cancer type, age, sex, comorbid depression, Medicare or commercial insurance, and dual eligibility for Medicare and Medicaid.

A multivariable step-wise logistic regression was constructed to assess factors associated with low comorbidity medication adherence. Covariates entered into the model included age, sex, higher comorbidity level, frequent mental unhealthy days, frequent physical unhealthy days, Medicare or commercial insurance, patient-reported cancer symptoms, and comorbid conditions with at least 10% prevalence in the study cohort. Results were reported as odds ratios with 95% confidence limits. All analyses were conducted using SAS Enterprise Guide version 7.1, with an a priori alpha of 0.05 to establish statistical significance.

## Results

Of 1847 eligible survey respondents, most were female with a mean (standard deviation) age of 69.2 (9.2) years. In a comparison of survey responders to non-responders, several differences were noted. Responders were older (69.7 vs. 68.3 years), and were more likely to be female (66.8% vs. 63.2%, *P* = 0.006) and white (84.9% vs. 81.4%). There was no difference by region or by level of comorbidity (Appendix 4).

Survey respondents predominantly resided in the Southern region of the United States, aligning with the geographic distribution of the Humana population (Table 1). Medicare Advantage patients comprised 88.2% of the respondents. Additionally, 11.8% were dually eligible for Medicare Advantage and Medicaid. More than half (57.2%) reported being married and 60.3% lived with a spouse or significant other at the time of the survey. Frequent overall unhealthy days were reported by 46.2% of the respondents; frequent physically unhealthy days by 34.9% of the survey respondents and frequent mentally unhealthy days were reported by 22.8% of the survey respondents.

**Table 1:** Demographics of survey respondents by level of comorbidity medication adherence

	Overall *N* = 1847	Low Adherence *n* = 508	Moderate/ High Adherence *n* = 1212	*P* value
Cancer type, n (%)				
Metastatic breast cancer	700 (37.9)	176 (34.7)	482 (39.8)	0.137
Metastatic colorectal cancer	524 (28.4)	153 (30.1)	336 (27.7)	
Metastatic lung cancer	623 (33.7)	179 (35.2)	394 (32.5)	
Demographic Characteristics				
Age, years, mean [SD]	69.2 [9.20]	68.5 [9.88]	69.5 [8.68]	0.043
Sex female, n (%)	1233 (66.8)	324 (63.8)	827 (68.2)	0.073
Race/Ethnicity (Medicare only), n (%)				
White	1379 (84.9)	373 (85.7)	910 (84)	0.767
Non-white	240 (13.0)	60 (11.8)	170 (14.0)	
Unknown	5 (0.3)	2 (0.5)	3 (0.3)	
Marital Status, n (%)				
Married	1051 (57.2)	298 (59.1)	679 (56.3)	0.558
Divorced	268 (14.6)	71 (14.1)	180 (14.9)	
Separated	24 (1.3)	6 (1.2)	15 (1.2)	
Widowed	368 (20.0)	91 (18.1)	256 (21.2)	
Living in a marriage-like relationship	49 (2.7)	17 (3.4)	28 (2.3)	
Single, Never Married	77 (4.2)	21 (4.2)	49 (4.1)	
Lives alone, n (%)	444 (24.0)	108 (21.3)	310 (25.6)	0.057
Geographic Region, n (%)				
Northeast	29 (1.6)	9 (1.8)	16 (1.3)	0.432
Midwest	453 (24.5)	114 (22.4)	306 (25.2)	
South	1220 (66.1)	340 (66.9)	801 (66.1)	
West	145 (7.9)	45 (8.9)	89 (7.3)	
Medicare Advantage (vs. Commercial), n (%)	1629 (88.2)	436 (85.8)	1087 (89.7)	0.022
Dual eligibility for Medicare and Medicaid	192 (11.8)	41 (9.4)	32 (12.5)	0.094
Clinical Characteristics				
Comorbid conditions (pre-index), n (%)				
Anemia	547 (29.6)	154 (30.3)	349 (28.8)	0.527
Anxiety	286 (15.5)	84 (16.5)	184 (15.2)	0.480
Ischemia	386 (20.9)	111 (21.9)	249 (20.5)	0.544
Cerebrovascular diseases	189 (10.2)	49 (9.6)	128 (10.6)	0.569
Depression	236 (128)	70 (13.8)	156 (12.9)	0.611
Hypertension	1253 (67.8)	310 (61)	877 (72.4)	< 0.001
Renal disease including ESRD	306 (16.6)	90 (17.7)	192 (15.8)	0.338
Diabetes Mellitus	502 (27.2)	122 (24)	354 (29.2)	0.028
Pneumonia	189 (10.2)	56 (11)	113 (9.3)	0.280
Comorbidity Index, median [IQR]		8 [4.5]	8 [1]	0.002
High deyo Charlson comorbidity score (score ≥ 7), n (%)	1404 (6.0)	368 (72.4)	944 (77.9)	0.015
Healthy Days				
Frequent overall unhealthy days, n (%)	823 (46.2)	245 (52.8)	531 (44.1)	0.001
Frequent mental unhealthy days, n (%)	401 (22.8)	139 (30.3)	241 (20.2)	< 0.001
Frequent physical unhealthy days, n (%)	614 (34.9)	179 (38.7)	399 (33.6)	0.049
Patient-reported excellent, very good or good health, n (%)	993 (55.9)	243 (52.4)	678 (56.4)	0.138
Physical/mental health frequently impeded activities, n (%)	518 (29.5)	161 (35.0)	329 (27.7)	0.004

Morisky Medication Adherence Score < 6 was defined as low comorbidity medication adherence

Morisky Medication Adherence Score = 6, 7 or 8 was defined as moderate/high medication adherence

Differences were assessed using a t-test for continuous variables and a chi-square test for categorical variables

Low adherence to comorbidity medication was reported by 29.5% of patients (Table 1). Patients reporting low comorbidity medication were younger, less frequently covered by a Medicare Advantage health plan, and had a lower comorbidity level than patients more adherent to their comorbidity medications. There were no differences in comorbidity medication adherence levels by cancer type.

Overall, hypertension was the most common comorbidity (67.8%). A greater proportion of patients with low comorbidity medication adherence had comorbid hypertension than patients with moderate/high comorbidity medication adherence. Anemia was the second most prevalent comorbidity (29.6%), but did not differ by comorbidity medication adherence level. Diabetes was observed in over a quarter of patients (27.2%), and was observed less frequently for patients with low comorbidity medication adherence as compared to patients with moderate/high medication adherence. Overall, 76.0% of patients had a high comorbidity level, based on the DCCI. Patients with a higher comorbidity level were more frequently moderately or highly adherent to their comorbidity medication regimens.

**Fig. 1:**
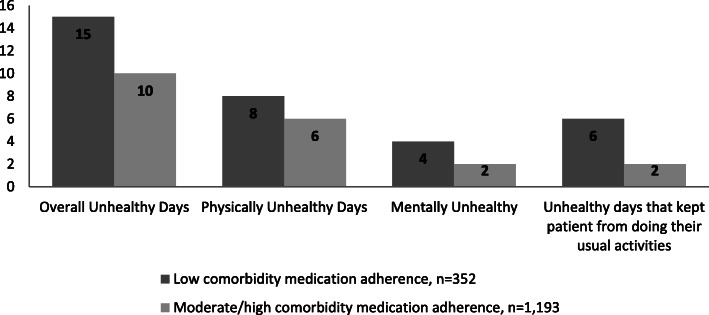
Median number of unhealthy days overall and by comorbidity medication adherence ^a^ **(**^a^All differences were statistically significant at the 0.05 level using the Wilcoxon rank sum test)

In unadjusted analysis testing the relationship between comorbidity medication adherence and HRQoL, low comorbidity medication adherence was found to be associated with significantly more total mental and physical unhealthy days (Fig. 1). Patients with low comorbidity medication adherence reported a median of 15 total unhealthy days compared to 10 total unhealthy days for patients with moderate or high comorbidity medication adherence (*P* < 0.05). Over half of patients with low levels of comorbidity medication adherence had frequent unhealthy days as compared to 44.1% for patients with moderate or high comorbidity medication adherence (*P* = 0.001). Patients with low comorbidity medication adherence reported both more frequent mental and physical unhealthy days than patients with moderate/high comorbidity medication adherence (30.3% vs 20.2%, *P* < 0.001; 38.7% vs 33.6%, *P* = 0.049). There was no difference in the patient-reported health status by level of comorbidity medication adherence; however, patients with low comorbidity medication adherence more frequently reported their mental or physical unhealthy days impeded their ability to perform their usual activities.

**Fig. 2:**
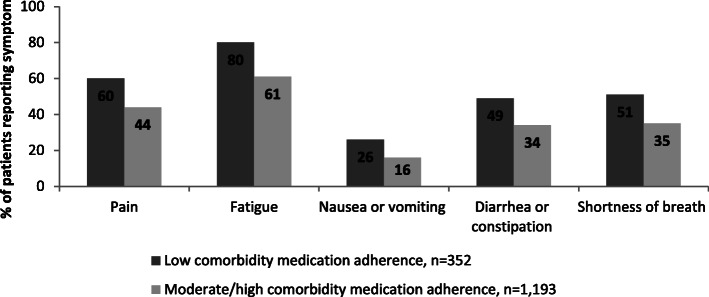
Patient-reported cancer-related symptoms by comorbidity medication adherence**.** Morisky Medication Adherence Score < 6 was defined as low comorbidity medication adherence. Morisky Medication Adherence Score = 6, 7 or 8 was defined as moderate/high medication adherence. All comparisons significant at the *P* < 0.001 level by chi-square test

A significantly greater proportion of patients with low comorbidity medication adherence reported experiencing pain, fatigue, nausea or vomiting, diarrhea or constipation and shortness of breath in the prior 30 days compared to patients with moderate/high comorbidity medication adherence in unadjusted analysis (Fig. 2).

Evaluation of the association between low comorbidity medication adherence and the number of unhealthy days, using a binomial Poisson regression model, revealed that patients reporting low comorbidity medication adherence had 23.4% more unhealthy days than patients with moderate/high comorbidity medication adherence controlling for other potentially confounding factors (*P* = 0.007, Table 2).

**Table 2:** Association between low comorbidity medication adherence and the number of unhealthy days for patients with metastatic breast, lung or colorectal cancer

Parameter	Incidence rate ratio	95% Confidence interval		*P* value
Breast cancer (compared to lung cancer)	0.813	0.676	0.978	0.028
Colorectal cancer (compared to lung cancer)	0.793	0.669	0.941	0.008
Age	0.898	0.782	1.033	0.132
Female	0.898	0.756	1.067	0.223
Low comorbidity medication adherence	1.234	1.059	1.439	0.007
Comorbid depression	1.551	1.315	1.830	< 0.001
High comorbidity index	0.963	0.819	1.132	0.647
Dual eligibility for Medicare and Medicaid	1.123	0.908	1.390	0.284

A negative binomial regression model was used to assess the association between low adherence to comorbidity medications and the number of unhealthy days among patients with metastatic breast, colorectal cancer and lung cancer while controlling for other potentially confounding variables

Using a multivariable logistic regression model to determine factors associated with low comorbidity medication adherence, a higher comorbidity index was the only factor observed as protective against the odds of low comorbidity medication adherence (Table 3). For patients with a high comorbidity index, the odds of low comorbidity medication adherence were reduced by 32.0%. Furthermore, patients reporting frequent mental unhealthy days had an 84.7% increased odds of low adherence to medication for their comorbidities. Some patient-reported cancer-related symptoms increased the odds of low adherence to comorbidity medications. Patients reporting fatigue had a 50.0% increased odds of low comorbidity adherence. Diarrhea or constipation was associated with a 45.5% increased odds and shortness of breath had a 39.9% increased odds of non-adherence with comorbidity medications.

**Table 3:** Correlates of low comorbidity medication adherence for patients with metastatic breast, lung or colorectal cancer

	Odds Ratio	95% Confidence Interval		*P* value
High comorbidity index	0.679	0.489	0.942	0.021
Frequent mental unhealthy days	1.857	1.341	2.572	< 0.001
Patient-reported fatigue	1.495	1.012	2.20	0.041
Patient-reported diarrhea/constipation	1.455	1.070	1.978	0.003
Patient-reported shortness of breath	1.399	1.015	1.930	0.005

A step-wise logistic regression with *P* = 0.05 entry criteria and *P* = 0.05 to remain in the model was used to evaluate factors associated with low comorbidity medication adherence among patients with metastatic breast, colorectal and lung cancer

Other variables considered in the model include cancer type, age, sex, anxiety, cardiovascular disease, chronic kidney disease, depression, endocrine disease, gastrointestinal disease, hematologic disease, osteoarthritis, pulmonary disease, hypertension, diabetes mellitus, Medicare or commercial insurance, low income subsidy, frequent physical unhealthy days, patient-reported pain, patient-reported fatigue and patient-reported nausea/vomiting

## Discussion

In this study, we observed that low adherence to comorbidity medications was negatively associated with HRQoL among mostly elderly patients with metastatic breast, lung or colorectal cancer. In adjusted analyses, patients with low comorbidity medication adherence experienced more than a 20% increase in unhealthy days compared to patients with moderate or high comorbidity medication adherence. A greater proportion of patients with low comorbidity medication adherence reported cancer-related symptoms or treatment side-effects as compared to patients who were more likely to be adherent to their comorbidity medication regimens. These symptoms, or treatment side-effects, could be related to the cancer, cancer treatment, the comorbidity or comorbidity treatment, or some combination of these factors.

Medication adherence for concomitant diseases can be particularly challenging for patients with cancer, and lack of adherence can compound health issues resulting in worsening of overall health [28]. This has been reported by a number of studies that explored factors that influence a patient’s level of adherence to medications for specific comorbidities [29–32]. For example, prior work has shown lower levels of adherence and persistence for medications for diabetes for women with breast cancer, as compared to a matched cohort of women without breast cancer; however, these differences were not observed for hypertensive therapy or lipid-lowering medications among the same population [32]. While this example compared adherence to comorbidity medication in women with or without breast cancer, in contrast, our study focused on adherence to non-cancer medications in a metastatic cancer cohort.

This study advances understanding of how to assist patients achieve higher HRQoL by demonstrating that low adherence to comorbidity medication was associated with worse HRQoL for patients with metastatic cancer. In fact, patients with low comorbidity medication adherence had five more unhealthy days over the course of a month as compared to patients who were more adherent. Our study is unique in its consideration of the broad effect of adherence to comorbid medications for patients undergoing cancer treatment.

We approached this study with the awareness that we did not have insight into whether or not adherence to medications for a specific comorbidity was different in the presence of cancer treatment. A surprising finding in our study was that patients with higher comorbidity levels were more frequently moderately or highly adherent to their comorbidity medication regimens than were patients with low comorbidity adherence levels. This may indicate that it is not the number of comorbidities, but perhaps the severity or degree to which the comorbidity is controlled that differentiates HRQoL. This relationship, though difficult to ascertain in claims data, warrants further consideration.

A factor that may help facilitate medication adherence is the identification of polypharmacy in patients with higher comorbidity levels, which can trigger referral to resources, such as disease management and medication adherence and reconciliation programs. This may support care coordination for other conditions while undergoing cancer treatment. If this assumption is correct, one implication is that oncology management programs may need to increase monitoring of patients with fewer, or less severe, comorbidities to be sure that compliance with medications for these comorbidities is not compromised while patients undergo treatment for their cancer. Or as noted by Santorellie et al., primary care coordination may be beneficial for patients with cancer [32].

Our study found an association between the presence of cancer-related symptoms and low adherence to comorbidity medication. Given the cross-sectional nature of this study, it is not possible to ascertain a causal pathway in the relationship. However, it is possible that patients are not compliant with comorbidity medication regimens because their cancer-related symptoms impede compliance or the comorbidity medications are contraindicated during cancer treatment. It is possible that noncompliance with comorbidity medication regimens may exacerbate cancer-related symptoms or mitigate control of those symptoms. Additional knowledge regarding the nature and direction of this association could give providers insights on how to encourage patient compliance with prescribed comorbidity medications while undergoing cancer treatment as a means of promoting or preserving HRQoL.

### Limitations

This study was conducted in an elderly cohort of patient, most of whom were insured through a Medicare Advantage health plan. The findings of this study may not be generalizable to younger or uninsured populations.

Though specific cancer-related measures of HRQoL have been validated and used in other studies [33], the Healthy Days measure was used in our study since the two questions that comprise the measure of HRQoL can be easily implemented in a clinical setting. The Healthy Days tool has certain limitations that warrant mention. As noted by Slabaugh et al., the Healthy Days survey questions were validated individually [34]. The use of both physical and mental unhealthy days jointly, as an indication of total unhealthy days, has not been validated. Also, in this study we capped total unhealthy days at 30. While this constructed variable is often used, it does somewhat prohibit our ability to distinguish patients with the worst HRQoL. Also, because patients may have habituated to their health status under ongoing cancer treatment, the responses may subject to a positive response shift.

This study did not control for the timing between the assessment of HRQoL and cancer treatment. Patients currently undergoing cancer treatment may have different levels of cancer-related symptoms and adherence to comorbidity medication than patients who recently completed treatment. Future studies should evaluate if there are differences in HRQoL for patients undergoing cancer treatment as compared to patients who have recently completed treatment. We expect that HRQoL fluctuates throughout the course of diagnosis, treatment and following treatment. This cross-sectional study did not explore variations in HRQoL over the trajectory of patients’ cancer experience.

Also, our use of the MMAS to capture adherence to medications for comorbidities in general, as opposed to adherence for a specific medication, has not been validated. Previous studies have found the MMAS to be highly correlated with medication adherence ascertained via other methods (pharmacy and medical claims, dispensing monitors) [23, 24], but it has not been validated for medications used to treat comorbidities [23, 35]. additionally, it is possible that patients had varying levels of adherence for different medications, and the use of a single scale may have masked these differences. Another limitation of the MMAS is that while the questions are related to the frequency of forgetting to take medications or inconvenience associated with taking medications besides one question that asks if patients stopped taking the medication because of how it made them feel, it does not question if there are deliberate reasons that a patient has chosen to not take their medication. Specifically, the questions do not ask the reasons for non-compliance, such as a deliberate choice not to take the medications because of symptoms, side effects, financial limitations, other barriers or because the patient made a conscious decision to not take their medication in the face of contending with cancer.

Finally, though the questions used to assess patients’ cancer-related symptoms were constructed in alignment with the format used in the MHOS, they have not been validated.

## Conclusion

The findings from this study suggest that HRQoL, as measured by the CDCs Healthy Days measurement tool, is worse for patients with metastatic cancer who have a lower level of compliance to their comorbidity medications. Our results suggest that increasing patients’ level of comorbidity medication adherence, perhaps by focusing on care coordination and/or medication therapy management during cancer treatment, or by consideration of comorbidities within the context of oncology management, may be an opportunity for improving or maintaining HRQoL for older patients with metastatic cancer.

## Appendix 1

**Table 4:** Baseline comorbidities and associated ICD-9-CM codes

Body system	Disease	Prevalent Chronic medications	ICD-9-CM Codes
Cardiovascular			
	hypertension	B-blockers, diuretics, Ca-channel blockers, ACE-I, ARBs	4011, 4019, 4010, 40,200, 40,201, 40,210, 40,211, 40,290, 40,291, 4030, 40,300, 40,301, 4031, 40,310, 40,311, 4039, 40,390, 40,391, 4040, 40,400, 40,401, 40,402, 40,403, 4041, 40,410, 40,411, 40,412, 40,413, 4049, 40,490, 40,491, 40,492, 40,493, 40,501, 40,509, 40,511, 40,519, 40,591, 40,599, 4372
	coronary artery disease	Aspirin, ranolazine,	4110, 4111, 4118, 41,181, 41,189, 412, 4130, 4131, 4139, 4140, 41,400, 41,401, 41,406, 4142, 4143, 4144, 4148, 4149, V4581, V4582
	congestive heart failure	B-blockers, diuretics, Ca-channel blockers, ACE-I, ARBs	39,891, 4280, 4281, 42,820, 42,821, 42,822, 42,823, 42,830, 42,831, 42,832, 42,833, 42,840, 42,841, 42,842, 42,843, 4289
	atrial fibrillation	B-blockers, warfarin, Factor-X inhibitors	42,731, 42,732
Pulmonary			
	chronic obstructive pulmonary disease	SABAs, LABAs, anticholinergics, inhaled corticosteroids	490, 4910, 4911, 4912, 49,120, 49,121, 49,122, 4918, 4919, 4920, 4928, 494, 4940, 4941, 496
	asthma	SABA, inhaled corticosteroids, anticholinergics	49,300, 49,301, 49,302, 49,310, 49,311, 49,312, 49,320, 49,321, 49,322, 49,381, 49,382, 49,390, 49,391, 49,392
Gastrointestinal			
	gastroesophageal reflux disease	H-1 receptor blockers, proton pump inhibitors	53,081
Hematologic			
	anemia	Folic acid, Iron, B-12, erythropoietin	2800, 2801, 2808, 2809, 2810, 2811, 2812, 2813, 2814, 2818, 2819, 2820, 2821, 2822, 2823, 2824, 28,240, 28,243, 28,244, 28,245, 28,246, 28,247, 28,249, 2827, 2828, 2829, 2830, 2831, 28,310, 28,311, 28,319, 2832, 2839, 2840, 28,401, 28,409, 2841, 28,412, 28,419, 2842, 2848, 28,481, 28,489, 2849, 2850, 28,521, 28,529, 2858, 2859, 2851
Endocrine/ metabolic			
	hypothyroidism	Levothyroxine, liothyronine	2449
	diabetes mellitus, type 2	Oral antidiabetic medications, insulin	24,900, 25,000, 25,001, 7902, 79,021, 79,022, 79,029, 7915, 7916, V4585, V5391, V6546, 24,901, 24,910, 24,911, 24,920, 24,921, 24,930, 24,931, 24,940, 24,941, 24,950, 24,951, 24,960, 24,961, 24,970, 24,971, 24,980, 24,981, 24,990, 24,991, 25,002, 25,003, 25,010, 25,011, 25,012, 25,013, 25,020, 25,021, 25,022, 25,023, 25,030, 25,031, 25,032, 25,033, 25,040, 25,041, 25,042, 25,043, 25,050, 25,051, 25,052, 25,053, 25,060, 25,061, 25,062, 25,063, 25,070, 25,071, 25,072, 25,073, 25,080, 25,081, 25,082, 25,083, 25,090, 25,091, 25,092, 25,093
	Hyperlipidemia, dyslipidemia, hypercholesterolemia	Statins, fibrates, niacin, cholystyramine	2720, 2721, 2722, 2723, 2724, 2720, 2721, 2722, 2723, 2724
Allergy/ rheumatology			
	allergic rhinitis	Intranasal corticosteroids, H1- blockers	4772, 4778, 4779
Genitourinary			
	chronic kidney disease	ACE-I, ARBs	585, 5851, 5852, 5853, 5854, 5855, 5856, 5859, 7925, V420, V451, V4511, V4512, V560, V561, V562, V5631, V5632, V568
Neurologic			
	seizure disorder	Anti-epileptic medication	3450, 34,500, 34,501, 3451, 34,510, 34,511, 3452, 3453, 3454, 34,540, 34,541, 3455, 34,550, 34,551, 3456, 34,560, 34,561, 3457, 34,570, 34,571, 3458, 34,580, 34,581, 3459, 34,590, 34,591, 7803, 78,031, 78,032, 78,033, 78,039
Psychiatric			
	depression	SSRi, tricyclic antidepressants	311
	anxiety	SSRi, tricyclic antidepressants, benzodiazepines	29,384, 30,000, 30,001, 30,002, 30,009, 30,010, 30,020, 30,021, 30,022, 30,023, 30,029, 3003, 3005, 30,089, 3009, 3080, 3081, 3082, 3083, 3084, 3089
Musculoskeletal			
	osteoarthritis	APAP, NSAIDS, opioids	71,500, 71,504, 71,509, 71,510, 71,511, 71,512, 71,513, 71,514, 71,515, 71,516, 71,517, 71,518, 71,520, 71,521, 71,522, 71,523, 71,524, 71,525, 71,526, 71,527, 71,528, 71,530, 71,531, 71,532, 71,533, 71,534, 71,535, 71,536, 71,537, 71,538, 71,580, 71,589, 71,590, 71,591, 71,592, 71,593, 71,594, 71,595, 71,596, 71,597, 71,598, V134

## Appendix 2

**Table 5:** Healthy Days Survey

Question	Response options
1. Would you say your general health is	Excellent Very good Good Fair Poor
2. Now thinking about your physical health, which includes physical illness and injury, for how many days during the past 30 days was your physical health not good?	Number of days
3. Now thinking about your mental health, which includes stress, depression, and problems with emotions, for how many days during the past 30 days was your mental health not good?	Number of days
4. During the past 30 days, for about how many days did poor physical or mental health keep you from doing your usual activities, such as self-care, work, or recreation?	Number of days

## Appendix 3

**Table 6:** Morisky Medication Adherence Scale

Question	Response options
1. Do you sometimes forget to take your medications?	Yes/No
2. People sometimes miss taking their medications for reasons other than forgetting. Over the past two weeks, were there any days when you did not take your medicine?	Yes/No
3. Have you ever cut back or stopped taking your medication without telling your doctor, because you felt worse when you took it?	Yes/No
4. When you travel or leave home, do you sometimes forget to bring your medication?	Yes/No
5. When you feel like your health conditions are under control, do you sometimes stop taking your medicine?	Yes/No
6. Taking medication every day is a real inconvenience for some people. Do you ever feel hassled about sticking to your treatment plan?	Yes/No
7. Did you take all your medicine yesterday?	Yes/No
8. How often do you have difficulty remembering to take all of your medications?	Never/rarely Once in a while Sometimes Usually All of the time

Use of the ©MMAS-8 is protected by US copyright laws. Permission for use is required. A licensure agreement is available from: Donald E. Morisky, ScD, ScM, MSPH, Professor, Department of Community Health Sciences, UCLA School of Public Health, 650 E.Young Drive, South, Los Angeles, CA 90095–1772

## Appendix 4

**Table 7:** Comparison of survey responders and non-responders

Demographic Characteristics	Overall *N* = 7435	Non-responders *n* = 5538 (75.2%)	Responders *n* = 1847 (25.8%)	*P* value
Cancer type, n(%)				
Breast cancer	2425 (32.6)	1725 (30.9)	700 (37.9)	< 0.001
Colorectal cancer	2109 (28.4)	1585 (28.4)	524 (28.4)	
Lung cancer	2901 (39)	2278 (40.8)	623 (33.7)	
Age, years (Mean, SD)	68.6+/−9.46	68.3+/−9.67	69.7+/−8.63	< 0.001
Gender (1 = female)	4768 (64.1)	3534 (63.2)	1234 (66.8)	0.006
Race/Ethnicity (Medicare only)				
White	5264 (82.3)	3885 (81.4)	1379 (84.9)	0.006
Black	918 (14.4)	717 (15)	201 (12.4)	.
Other	213 (3.3)	169 (3.5)	44 (2.7)	.
Geographic Region				
Northeast	108 (1.5)	79 (1.4)	29 (1.6)	0.326
Midwest	1759 (23.7)	1306 (23.4)	453 (24.5)	
South	5033 (67.7)	3813 (68.2)	1220 (66.1)	
West	535 (7.2)	390 (7)	145 (7.9)	
Plan Type = Medicare Advantage (vs. Commercial)	6380 (85.8)	4761 (85.2)	1619 (87.7)	0.009
Dual Eligible	937 (14.7)	745 (15.6)	192 (11.8)	< 0.001
Clinical Characteristics				
Comorbidity Index	7.66+/−3.24	7.66+/−3.28	7.68+/−3.13	0.834
Comorbid conditions (pre-index)				
Anemia	2302 (31)	1755 (31.4)	547 (29.6)	0.149
Anxiety	1294 (17.4)	1008 (18)	286 (15.5)	0.012
Autoimmune disorders	590 (7.9)	445 (8.0)	145 (7.9)	0.876
Ischemia	1616 (21.7)	1230 (22)	386 (20.9)	0.315
Cardiac diseases	29 (0.4)	24 (0.4)	5 (0.3)	0.343
Cerebrovascular diseases	864 (11.6)	675 (12.1)	189 (10.2)	0.032
Depression	1079 (14.5)	843 (15.1)	236 (12.8)	0.015
Hypertension	5023 (67.6)	3770 (67.5)	1253 (67.8)	0.766
Liver diseases	38 (0.5)	28 (0.5)	10 (0.5)	0.833
Pulmonary disease	229 (3.1)	169 (3)	60 (3.2)	0.629
Renal disease including ESRD	1264 (17)	958 (17.1)	306 (16.6)	0.568
Rheumatoid arthritis	214 (2.9)	166 (3)	48 (2.6)	0.407
Diabetes Mellitus	2210 (29.7)	1708 (30.6)	502 (27.2)	0.006
Pneumonia	859 (11.6)	670 (12.0)	189 (10.2)	0.041
Osteoporosis	577 (7.8)	430 (7.7)	147 (8.0)	0.713
