# Correction to: The relationship between comorbidity medication adherence and health related quality of life among patients with cancer

**DOI:** 10.1186/s41687-021-00300-6

**Published:** 2021-03-09

**Authors:** Dana Drzayich Antol, Adrianne Waldman Casebeer, Raya Khoury, Todd Michael, Andrew Renda, Sari Hopson, Aparna Parikh, Alisha Stein, Mary Costantino, Stephen Stemkowski, Mikele Bunce

**Affiliations:** 1 Louisville, KY USA; 2 South San Francisco, California USA; 3 Humana Inc, Louisville, KY USA

**Correction to: J Patient-Rep Outcomes 2, 29 (2018)**

**https://doi.org/10.1186/s41687-018-0057-2**

Following publication of the original article [1], some errors were identified in relation to the Morisky Medication Adherence Scale (MMAS-8).

- The legend related to Table 1 should read:

Morisky Medication Adherence Score < 6 was defined as low comorbidity medication adherence.

Morisky Medication Adherence Score = 6–8 was defined as moderate/high medication adherence.

Differences were assessed using a t-test for continuous variables and a chi-square test for categorical variables.

-The legend related to Fig. 2 should read:

Morisky Medication Adherence Score < 6 was defined as low comorbidity medication adherence.

Morisky Medication Adherence Score = 6–8 was defined as moderate/high medication adherence.

Differences were assessed using a t-test for continuous variables and a chi-square test for categorical variables.

-In the reference section, one additional reference should be added after reference 24:Morisky DE, DiMatteo MR. Improving the measurement of self-reported medication nonadherence: Final response. J Clin Epidemio 2011; 64:258–263. PMID:21144706.

The authors regret any error regarding the Morisky Medication Adherence Scale in the original publication.
